# Crystal structure of poly[di-μ-aqua-{5-[(1*Z*)-2-(4-chloro­phen­yl)-1-cyano­ethenyl]-1,2,3,4-tetra­zol-1-ido-κ*N* ^1^}sodium]

**DOI:** 10.1107/S2056989015006325

**Published:** 2015-04-09

**Authors:** Joel T. Mague, Shaaban K. Mohamed, Mehmet Akkurt, Ahmed M. M. El-Saghier, Mustafa R. Albayati

**Affiliations:** a Department of Chemistry, Tulane University, New Orleans, LA 70118, USA; b Chemistry and Environmental Division, Manchester Metropolitan University, Manchester M1 5GD, England; c Chemistry Department, Faculty of Science, Minia University, 61519 El-Minia, Egypt; d Department of Physics, Faculty of Sciences, Erciyes University, 38039 Kayseri, Turkey; e Department of Chemistry, Faculty of Science, Sohag University, Sohag 82524, Egypt; f Kirkuk University, College of Science, Department of Chemistry, Kirkuk, Iraq

**Keywords:** crystal structure, sodium salt, tetra­zoles, hydrogen bonding

## Abstract

In the title compound, [Na(C_10_H_5_ClN_5_)(H_2_O)_2_]_*n*_, infinite chains of [Na(H_2_O)_2_]^+^ cations having a diamond-shaped cross-section and running parallel to the *b* axis are formed. O—H⋯N hydrogen bonds to the anions generate layers parallel to (100) which have the chloro­benzene­cyano­ethenyl substituents protruding from both surfaces. The sodium ion makes a short contact of 2.4801 (13) Å with the N atom of the tetra­zolide ring which is *syn* to the cyano N atom.

## Related literature   

For chemical behaviour of tetra­zoles, see: Smith *et al.* (1991 ▸); Duncia *et al.* (1990 ▸). For various industrial applications of different tetra­zole derivatives, see: Modarresi *et al.* (2009 ▸); Singh *et al.* (1980 ▸). For medicinal activities of compounds with a tetra­zole scaffold, see: Myznikov *et al.* (2007 ▸); Schocken *et al.* (1989 ▸); Mekni & Bakloiti (2008 ▸); Lim *et al.* (2007 ▸).
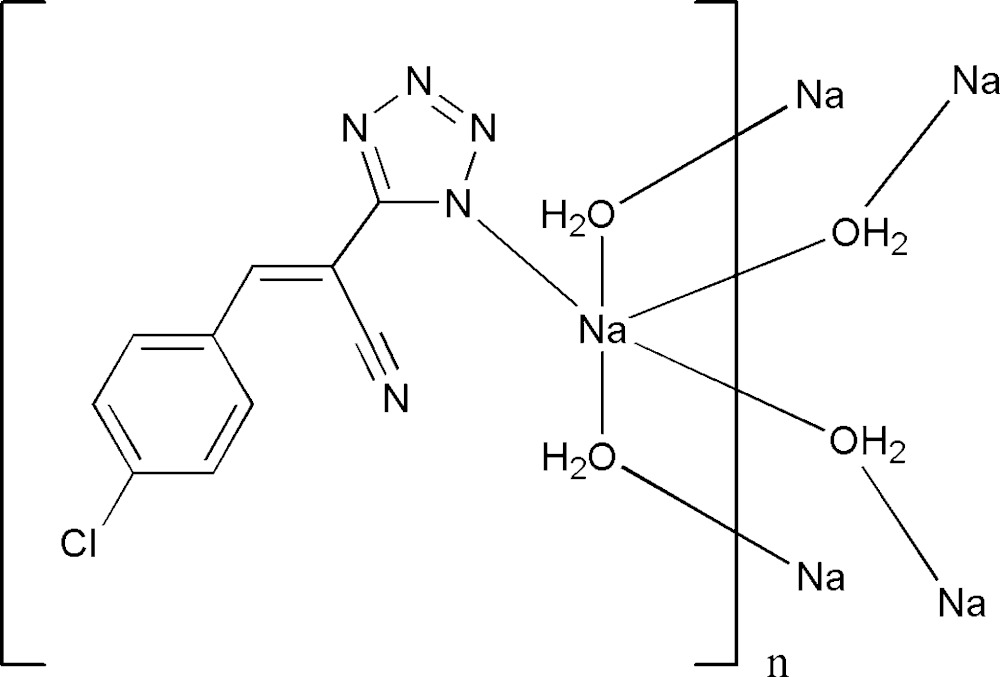


## Experimental   

### Crystal data  

[Na(C_10_H_5_ClN_5_)(H_2_O)_2_]
*M* *_r_* = 289.66
Monoclinic, 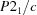

*a* = 22.0438 (4) Å
*b* = 3.8343 (1) Å
*c* = 15.0141 (3) Å
β = 92.427 (1)°
*V* = 1267.89 (5) Å^3^
*Z* = 4
Cu *K*α radiation
μ = 3.08 mm^−1^
*T* = 150 K
0.29 × 0.11 × 0.04 mm

### Data collection  

Bruker D8 VENTURE PHOTON 100 CMOS diffractometer
Absorption correction: multi-scan (*SADABS*; Bruker, 2014 ▸) *T*_min_ = 0.73, *T*_max_ = 0.89
9115 measured reflections
2560 independent reflections
2249 reflections with *I* > 2σ(*I*)
*R*_int_ = 0.026

### Refinement  

*R*[*F* ^2^ > 2σ(*F* ^2^)] = 0.032
*wR*(*F* ^2^) = 0.086
*S* = 1.03
2560 reflections
172 parameters
H-atom parameters constrained
Δρ_max_ = 0.36 e Å^−3^
Δρ_min_ = −0.37 e Å^−3^

### 

Data collection: *APEX2* (Bruker, 2014 ▸); cell refinement: *SAINT* (Bruker, 2014 ▸); data reduction: *SAINT*; program(s) used to solve structure: *SHELXT* (Sheldrick, 2015*a* ▸); program(s) used to refine structure: *SHELXL2014* (Sheldrick, 2015*b* ▸); molecular graphics: *DIAMOND* (Brandenburg & Putz, 2012 ▸); software used to prepare material for publication: *SHELXTL* (Sheldrick, 2008 ▸).

## Supplementary Material

Crystal structure: contains datablock(s) global, I. DOI: 10.1107/S2056989015006325/tk5364sup1.cif

Structure factors: contains datablock(s) I. DOI: 10.1107/S2056989015006325/tk5364Isup2.hkl

**Fig. 1.**
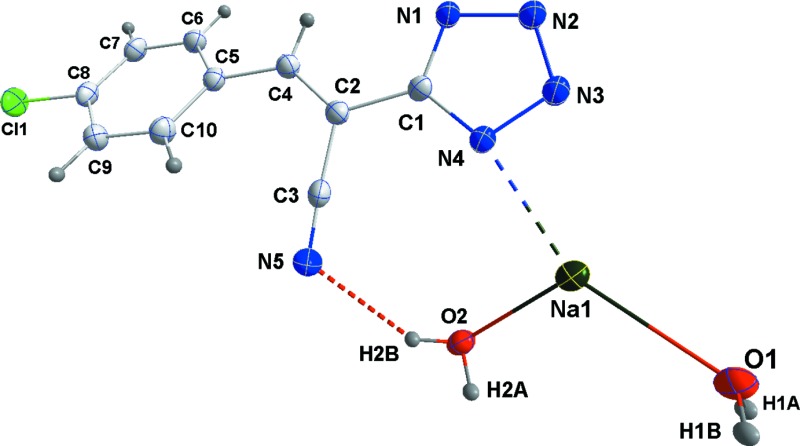
Title compound with numbering scheme and 50% probability ellipsoids.

. DOI: 10.1107/S2056989015006325/tk5364fig1.tif
Title compound with numbering scheme and 50% probability ellipsoids.

**Fig. 2.**
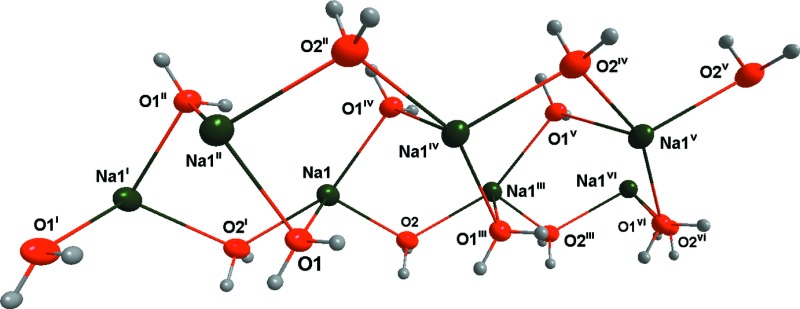
A portion of the {[Na(H2O)2]+}n chain (symmetry operations: (i) x, 1 + y, z, (ii) 2 - x, 1/2 + y, 3/2 - z, (iii) x, -1 + y, z, (iv) 2 - x, -1/2 + y, 3/2 - z, (v) 2 - x, -3/2 + y, 3/2 - z, (vi) x, -2 + y, z).

**Fig. 3.**
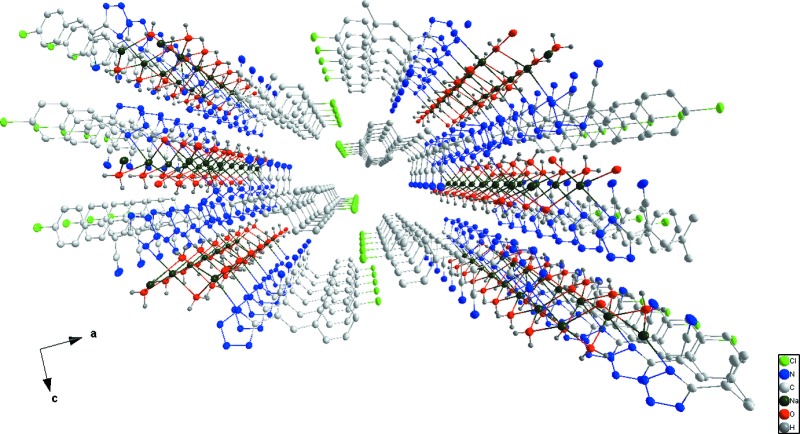
Packing viewed along the b axis.

Click here for additional data file.2 2 + n x y z x y z x y z x y z x y z x y z . DOI: 10.1107/S2056989015006325/tk5364fig2.tif
A portion of the {[Na(H_2_O)_2_]^+^}_n_ chain (symmetry operations: (i) *x*, 1 + *y*, *z*, (ii) 2 − *x*, 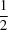 + *y*, 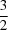 − *z*, (iii) *x*, −1 + *y*, *z*, (iv) 2 − *x*, −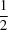 + *y*, 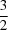 − *z*, (v) 2 − *x*, −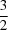 + *y*, 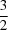 − *z*, (vi) *x*, −2 + *y*, *z*).

b . DOI: 10.1107/S2056989015006325/tk5364fig3.tif
Packing viewed along the *b* axis.

**Fig. 4.**
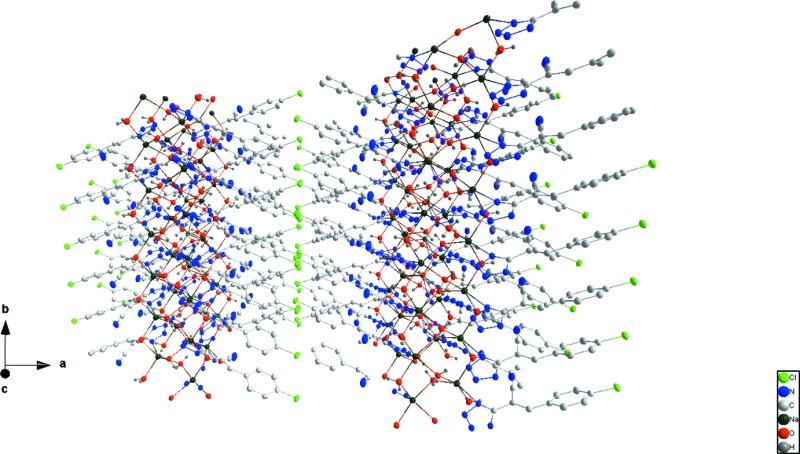
Elevation view of the chain structure.

. DOI: 10.1107/S2056989015006325/tk5364fig4.tif
Elevation view of the chain structure.

CCDC reference: 1056677

Additional supporting information:  crystallographic information; 3D view; checkCIF report

## Figures and Tables

**Table 1:** Hydrogen-bond geometry (, )

*D*H*A*	*D*H	H*A*	*D* *A*	*D*H*A*
O1H1*A*N3^i^	0.84	2.04	2.8443(17)	160
O1H1*B*N2^ii^	0.84	2.02	2.8593(17)	175
O2H2*B*N5	0.84	2.44	3.1009(19)	136

Symmetry codes: (i) 
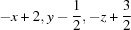
; (ii) 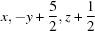.

## supplementary crystallographic information

### S1. Comment

Tetrazole compounds have been largely associated with a wide range of applications in medicine, biochemistry and agriculture (Modarresi *et al.*, 2009; Singh *et al.*, 1980). The medicinal activity of the tetrazole functionality is due to its ability to serve as a bioequivalent (bioisostere) of the carboxylic acid group (Smith *et al.*, 1991; Duncia *et al.*, 1990). They are also used as anti-hypertensive, anti-allergic, anti-biotic and anti-convulsant agents (Myznikov *et al.*, 2007; Schocken *et al.*, 1989; Mekni & Bakloiti, 2008; Lim *et al.*, 2007). As part of our on-going study of bio-active molecules we herein report the synthesis and X-ray structure of the title compound as a building block precursor in the synthesis of new tetrazole scaffold compounds.

The title compound, Fig. 1, comprises infinite chains of [Na(H_2_O)_2_]^+^ cations having a diamond-shaped cross-section (Fig. 2) and running parallel to the *b* axis. These are associated on all four sides by tetrazoluide anions *via* O—H···N hydrogen bonds (Table 1) to generate layers parallel to (100) which have the chlorobenzenecyanoethenyl substituents protruding from both surfaces (Figs 3 and 4). Additionally, the sodium ion makes a contact of 2.4801 (13) Å with N4 of the tetrazolide ring which is significantly shorter than the sum of the ionic radius of Na^+^ and the van der Waals radius of N (2.71 Å). The C—N and N—N bond lengths in the ring (1.314 (2)–1.345 (2) Å) suggest significant delocalization of the negative charge. The hydrogen bonding interactions may restrict the cation to approach this site as opposed to the face of the ring. The tetrazolide and benzene rings, respectively, make dihedral angle of 4.8 (2) and 25.8 (2)° with the plane defined by C2–C4.

### S2. Experimental

The title compound has been obtained as an unexpected product from a multi-component reaction of 1 mmol (94 mg) of amino-pyridine, 4-chloro-benzaldehyde (1 mmol, 141.5 mg), malononitrile (1 mmol, 66 mg), sodium acetate (0.15 mmol, 12.3 mg) and sodium azide (1 mmol, 65 mg). The reaction mixture was refluxed in ethanol/water (1:1) and monitored by TLC until completion after 3 hours. On cooling, the solid precipitate was collected, dried under vacuum and recrystallized from ethanol to afford suitable crystals for X-ray diffraction.

### S3. Refinement

H-atoms attached to carbon were placed in calculated positions (C—H = 0.95 Å) while those attached to oxygen were placed in locations derived from a difference map and their parameters adjusted to give O—H = 0.84 Å. All were included as riding contributions with isotropic displacement parameters 1.2 times those of the attached atoms.

### Crystal data

**Table:**

[Na(C_10_H_5_ClN_5_)(H_2_O)_2_]	*F*(000) = 592
*M**_r_* = 289.66	*D*_x_ = 1.517 Mg m^−^^3^
Monoclinic, *P*2_1_/*c*	Cu *K*α radiation, λ = 1.54178 Å
*a* = 22.0438 (4) Å	Cell parameters from 6311 reflections
*b* = 3.8343 (1) Å	θ = 4.0–74.5°
*c* = 15.0141 (3) Å	µ = 3.08 mm^−^^1^
β = 92.427 (1)°	*T* = 150 K
*V* = 1267.89 (5) Å^3^	Plate, colourless
*Z* = 4	0.29 × 0.11 × 0.04 mm

### Data collection

**Table:**

Bruker D8 VENTURE PHOTON 100 CMOS diffractometer	2560 independent reflections
Radiation source: INCOATEC IµS micro–focus source	2249 reflections with *I* > 2σ(*I*)
Mirror monochromator	*R*_int_ = 0.026
Detector resolution: 10.4167 pixels mm^-1^	θ_max_ = 74.5°, θ_min_ = 2.0°
ω scans	*h* = −27→25
Absorption correction: multi-scan (*SADABS*; Bruker, 2014)	*k* = −4→4
*T*_min_ = 0.73, *T*_max_ = 0.89	*l* = −18→18
9115 measured reflections	

### Refinement

**Table:**

Refinement on *F*^2^	Primary atom site location: structure-invariant direct methods
Least-squares matrix: full	Secondary atom site location: difference Fourier map
*R*[*F*^2^ > 2σ(*F*^2^)] = 0.032	Hydrogen site location: mixed
*wR*(*F*^2^) = 0.086	H-atom parameters constrained
*S* = 1.03	*w* = 1/[σ^2^(*F*_o_^2^) + (0.0487*P*)^2^ + 0.4982*P*] where *P* = (*F*_o_^2^ + 2*F*_c_^2^)/3
2560 reflections	(Δ/σ)_max_ = 0.001
172 parameters	Δρ_max_ = 0.36 e Å^−^^3^
0 restraints	Δρ_min_ = −0.37 e Å^−^^3^

### Special details

**Table:**

Geometry. All e.s.d.'s (except the e.s.d. in the dihedral angle between two l.s. planes) are estimated using the full covariance matrix. The cell e.s.d.'s are taken into account individually in the estimation of e.s.d.'s in distances, angles and torsion angles; correlations between e.s.d.'s in cell parameters are only used when they are defined by crystal symmetry. An approximate (isotropic) treatment of cell e.s.d.'s is used for estimating e.s.d.'s involving l.s. planes.
Refinement. Refinement of *F*^2^ against ALL reflections. The weighted *R*-factor *wR* and goodness of fit *S* are based on *F*^2^, conventional *R*-factors *R* are based on *F*, with *F* set to zero for negative *F*^2^. The threshold expression of *F*^2^ > σ(*F*^2^) is used only for calculating *R*-factors(gt) *etc*. and is not relevant to the choice of reflections for refinement. *R*-factors based on *F*^2^ are statistically about twice as large as those based on *F*, and *R*- factors based on ALL data will be even larger. H-atoms attached to carbon were placed in calculated positions (C—H = 0.95 Å) while those attached to oxygen were placed in locations derived from a difference map and their parameters adjusted to give O—H = 0.84 Å. All were included as riding contributions with isotropic displacement parameters 1.2 times those of the attached atoms.

### Fractional atomic coordinates and isotropic or equivalent isotropic displacement parameters (Å^2^)

**Table:**

	*x*	*y*	*z*	*U*_iso_*/*U*_eq_	
Cl1	0.49516 (2)	0.10717 (11)	0.35718 (3)	0.03137 (13)	
N1	0.86367 (6)	1.0318 (4)	0.43087 (8)	0.0206 (3)	
N2	0.91834 (6)	1.1473 (4)	0.46047 (8)	0.0220 (3)	
N3	0.92611 (6)	1.0749 (4)	0.54572 (8)	0.0221 (3)	
N4	0.87680 (6)	0.9091 (4)	0.57424 (8)	0.0209 (3)	
N5	0.74923 (7)	0.5087 (5)	0.65661 (10)	0.0382 (4)	
C1	0.83943 (7)	0.8871 (4)	0.50194 (9)	0.0176 (3)	
C2	0.77918 (7)	0.7259 (4)	0.50140 (9)	0.0194 (3)	
C3	0.76111 (7)	0.6066 (5)	0.58710 (10)	0.0242 (3)	
C4	0.74473 (7)	0.6828 (4)	0.42591 (10)	0.0210 (3)	
H4	0.7629	0.7579	0.3729	0.025*	
C5	0.68365 (7)	0.5378 (4)	0.41324 (10)	0.0204 (3)	
C6	0.66615 (7)	0.4117 (4)	0.32820 (10)	0.0240 (3)	
H6	0.6941	0.4214	0.2818	0.029*	
C7	0.60884 (7)	0.2734 (4)	0.31088 (10)	0.0244 (3)	
H7	0.5977	0.1835	0.2535	0.029*	
C8	0.56795 (7)	0.2681 (4)	0.37836 (11)	0.0232 (3)	
C9	0.58373 (7)	0.3933 (5)	0.46302 (10)	0.0254 (3)	
H9	0.5553	0.3870	0.5088	0.030*	
C10	0.64131 (7)	0.5273 (4)	0.47993 (10)	0.0243 (3)	
H10	0.6523	0.6135	0.5377	0.029*	
Na1	0.92711 (3)	0.78850 (17)	0.72191 (4)	0.02362 (16)	
O1	0.99226 (5)	0.9704 (3)	0.84267 (6)	0.0211 (2)	
H1A	1.0146	0.8147	0.8659	0.025*	
H1B	0.9717	1.0768	0.8799	0.025*	
O2	0.86926 (5)	0.2859 (3)	0.75583 (7)	0.0251 (3)	
H2A	0.8664	0.3340	0.8101	0.030*	
H2B	0.8326	0.2395	0.7434	0.030*	

### Atomic displacement parameters (Å^2^)

**Table:**

	*U*^11^	*U*^22^	*U*^33^	*U*^12^	*U*^13^	*U*^23^
Cl1	0.0220 (2)	0.0323 (2)	0.0393 (2)	−0.00816 (16)	−0.00439 (15)	−0.00037 (17)
N1	0.0179 (6)	0.0247 (7)	0.0192 (6)	−0.0011 (5)	0.0002 (5)	0.0004 (5)
N2	0.0194 (6)	0.0248 (7)	0.0216 (6)	−0.0015 (5)	0.0001 (5)	0.0001 (5)
N3	0.0196 (6)	0.0251 (7)	0.0214 (6)	−0.0024 (5)	−0.0017 (5)	−0.0011 (5)
N4	0.0190 (6)	0.0243 (7)	0.0191 (6)	−0.0017 (5)	−0.0008 (5)	−0.0003 (5)
N5	0.0268 (8)	0.0584 (11)	0.0292 (7)	−0.0056 (8)	−0.0005 (6)	0.0161 (7)
C1	0.0171 (7)	0.0178 (7)	0.0179 (6)	0.0020 (6)	0.0007 (5)	−0.0010 (5)
C2	0.0181 (7)	0.0181 (7)	0.0221 (7)	0.0018 (6)	0.0019 (5)	0.0021 (6)
C3	0.0161 (7)	0.0301 (9)	0.0260 (8)	−0.0015 (6)	−0.0029 (6)	0.0041 (7)
C4	0.0195 (7)	0.0214 (7)	0.0222 (7)	0.0002 (6)	0.0022 (5)	0.0008 (6)
C5	0.0187 (7)	0.0187 (7)	0.0237 (7)	0.0013 (6)	−0.0009 (5)	0.0008 (6)
C6	0.0226 (8)	0.0262 (8)	0.0230 (7)	0.0024 (7)	0.0007 (6)	0.0003 (6)
C7	0.0243 (8)	0.0235 (8)	0.0250 (7)	0.0014 (7)	−0.0049 (6)	−0.0027 (6)
C8	0.0190 (7)	0.0190 (8)	0.0311 (8)	−0.0018 (6)	−0.0039 (6)	0.0020 (6)
C9	0.0201 (8)	0.0300 (9)	0.0263 (7)	0.0005 (7)	0.0028 (6)	0.0013 (7)
C10	0.0232 (8)	0.0262 (8)	0.0233 (7)	0.0001 (7)	−0.0014 (6)	−0.0028 (6)
Na1	0.0246 (3)	0.0255 (3)	0.0205 (3)	0.0022 (3)	−0.0020 (2)	−0.0011 (2)
O1	0.0211 (5)	0.0246 (6)	0.0176 (5)	0.0045 (4)	0.0001 (4)	−0.0001 (4)
O2	0.0252 (6)	0.0323 (6)	0.0175 (5)	0.0033 (5)	−0.0022 (4)	−0.0019 (5)

### Geometric parameters (Å, º)

**Table:**

Cl1—C8	1.7359 (16)	C7—C8	1.384 (2)
N1—C1	1.3343 (19)	C7—H7	0.9500
N1—N2	1.3419 (18)	C8—C9	1.389 (2)
N2—N3	1.3138 (17)	C9—C10	1.383 (2)
N3—N4	1.3449 (18)	C9—H9	0.9500
N4—C1	1.3374 (19)	C10—H10	0.9500
N4—Na1	2.4801 (13)	Na1—O2^i^	2.3619 (13)
N5—C3	1.150 (2)	Na1—O1	2.3697 (12)
C1—C2	1.465 (2)	Na1—O2	2.3780 (14)
C2—C4	1.348 (2)	Na1—O1^ii^	2.3941 (12)
C2—C3	1.438 (2)	O1—Na1^iii^	2.3941 (12)
C4—C5	1.462 (2)	O1—H1A	0.8399
C4—H4	0.9500	O1—H1B	0.8398
C5—C10	1.398 (2)	O2—Na1^iv^	2.3619 (13)
C5—C6	1.404 (2)	O2—H2A	0.8399
C6—C7	1.385 (2)	O2—H2B	0.8401
C6—H6	0.9500		
			
C1—N1—N2	104.89 (12)	C9—C10—C5	120.98 (15)
N3—N2—N1	109.33 (12)	C9—C10—H10	119.5
N2—N3—N4	109.65 (12)	C5—C10—H10	119.5
C1—N4—N3	104.47 (12)	O2^i^—Na1—O1	84.99 (4)
C1—N4—Na1	161.92 (11)	O2^i^—Na1—O2	107.99 (5)
N3—N4—Na1	92.06 (8)	O1—Na1—O2	112.82 (4)
N1—C1—N4	111.66 (13)	O2^i^—Na1—O1^ii^	156.29 (5)
N1—C1—C2	124.39 (13)	O1—Na1—O1^ii^	91.35 (4)
N4—C1—C2	123.95 (13)	O2—Na1—O1^ii^	95.05 (4)
C4—C2—C3	123.10 (14)	O2^i^—Na1—N4	79.46 (4)
C4—C2—C1	122.35 (13)	O1—Na1—N4	149.61 (5)
C3—C2—C1	114.51 (13)	O2—Na1—N4	96.83 (4)
N5—C3—C2	177.07 (17)	O1^ii^—Na1—N4	92.55 (4)
C2—C4—C5	129.74 (14)	O2^i^—Na1—N3	84.63 (4)
C2—C4—H4	115.1	O1—Na1—N3	125.07 (5)
C5—C4—H4	115.1	O2—Na1—N3	121.70 (4)
C10—C5—C6	118.38 (14)	O1^ii^—Na1—N3	78.35 (4)
C10—C5—C4	123.85 (14)	N4—Na1—N3	27.99 (4)
C6—C5—C4	117.74 (14)	Na1—O1—Na1^iii^	106.04 (4)
C7—C6—C5	121.07 (14)	Na1—O1—H1A	115.7
C7—C6—H6	119.5	Na1^iii^—O1—H1A	95.6
C5—C6—H6	119.5	Na1—O1—H1B	109.0
C8—C7—C6	119.03 (14)	Na1^iii^—O1—H1B	116.9
C8—C7—H7	120.5	H1A—O1—H1B	113.0
C6—C7—H7	120.5	Na1^iv^—O2—Na1	107.98 (5)
C7—C8—C9	121.26 (15)	Na1^iv^—O2—H2A	116.7
C7—C8—Cl1	119.77 (12)	Na1—O2—H2A	95.3
C9—C8—Cl1	118.96 (12)	Na1^iv^—O2—H2B	107.7
C10—C9—C8	119.26 (15)	Na1—O2—H2B	130.0
C10—C9—H9	120.4	H2A—O2—H2B	98.7
C8—C9—H9	120.4		
			
C1—N1—N2—N3	−0.19 (17)	C3—C2—C4—C5	4.3 (3)
N1—N2—N3—N4	0.17 (17)	C1—C2—C4—C5	−178.16 (15)
N1—N2—N3—Na1	31.8 (5)	C2—C4—C5—C10	23.5 (3)
N2—N3—N4—C1	−0.08 (17)	C2—C4—C5—C6	−158.45 (17)
N2—N3—N4—Na1	172.51 (11)	C10—C5—C6—C7	−1.3 (2)
N2—N1—C1—N4	0.14 (17)	C4—C5—C6—C7	−179.43 (15)
N2—N1—C1—C2	179.72 (14)	C5—C6—C7—C8	1.5 (2)
N3—N4—C1—N1	−0.04 (17)	C6—C7—C8—C9	−1.0 (2)
Na1—N4—C1—N1	−155.5 (3)	C6—C7—C8—Cl1	178.18 (13)
N3—N4—C1—C2	−179.62 (14)	C7—C8—C9—C10	0.3 (3)
Na1—N4—C1—C2	24.9 (4)	Cl1—C8—C9—C10	−178.91 (13)
N1—C1—C2—C4	6.0 (2)	C8—C9—C10—C5	−0.1 (3)
N4—C1—C2—C4	−174.48 (15)	C6—C5—C10—C9	0.6 (2)
N1—C1—C2—C3	−176.27 (15)	C4—C5—C10—C9	178.56 (15)
N4—C1—C2—C3	3.3 (2)		

Symmetry codes: (i) *x*, *y*+1, *z*; (ii) −*x*+2, *y*−1/2, −*z*+3/2; (iii) −*x*+2, *y*+1/2, −*z*+3/2; (iv) *x*, *y*−1, *z*.

### Hydrogen-bond geometry (Å, º)

**Table:**

*D*—H···*A*	*D*—H	H···*A*	*D*···*A*	*D*—H···*A*
O1—H1*A*···N3^ii^	0.84	2.04	2.8443 (17)	160
O1—H1*B*···N2^v^	0.84	2.02	2.8593 (17)	175
O2—H2*B*···N5	0.84	2.44	3.1009 (19)	136

Symmetry codes: (ii) −*x*+2, *y*−1/2, −*z*+3/2; (v) *x*, −*y*+5/2, *z*+1/2.
