# Supplementary figures and images for: Crystal structure of poly[di-μ-aqua-{5-[(1Z)-2-(4-chloro­phen­yl)-1-cyano­ethenyl]-1,2,3,4-tetra­zol-1-ido-κN 1}sodium]
Source: Acta Crystallogr E Crystallogr Commun. 2015 Apr 9;71(Pt 5):m102–3. doi: 10.1107/S2056989015006325 (PMC4420058; doi:10.1107/S2056989015006325)

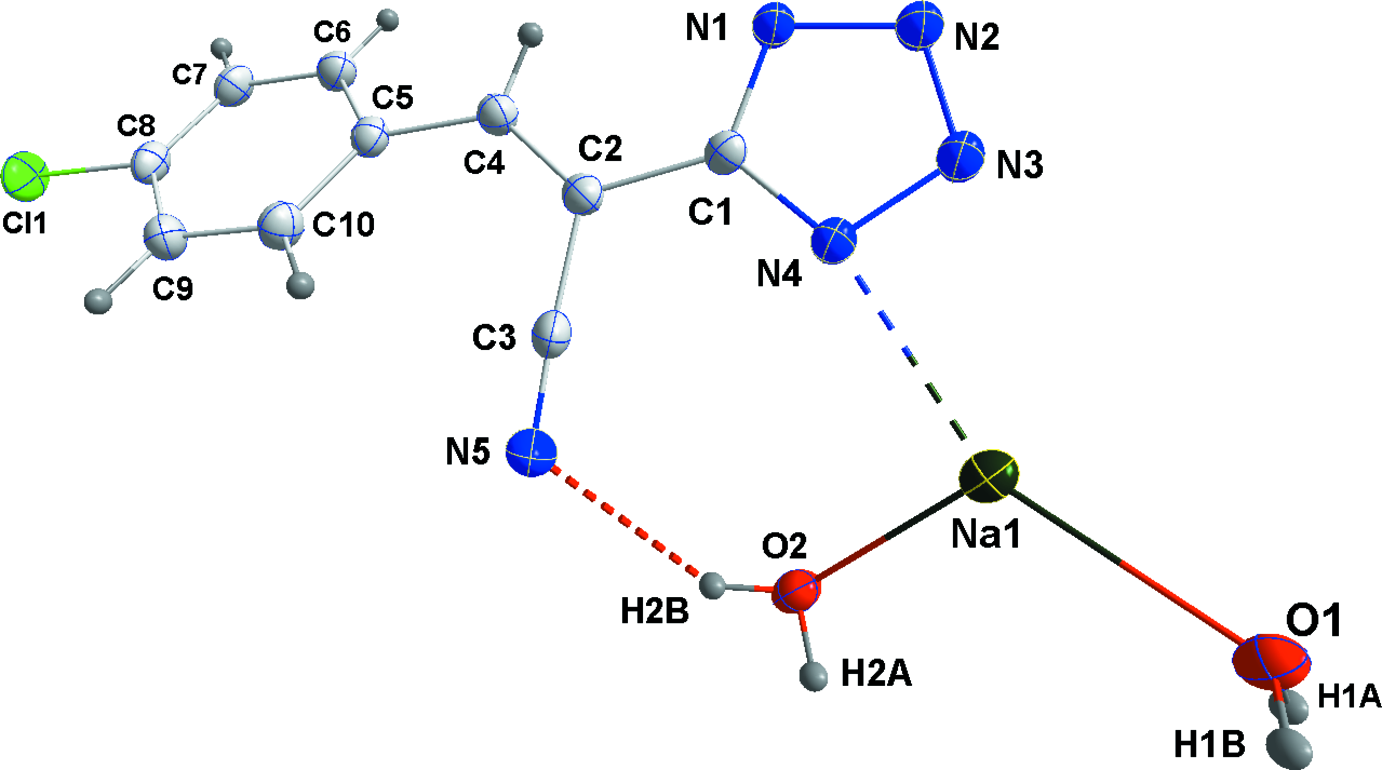

Supplement: Supplementary file 3 [file e-71-0m102-fig1.tif]

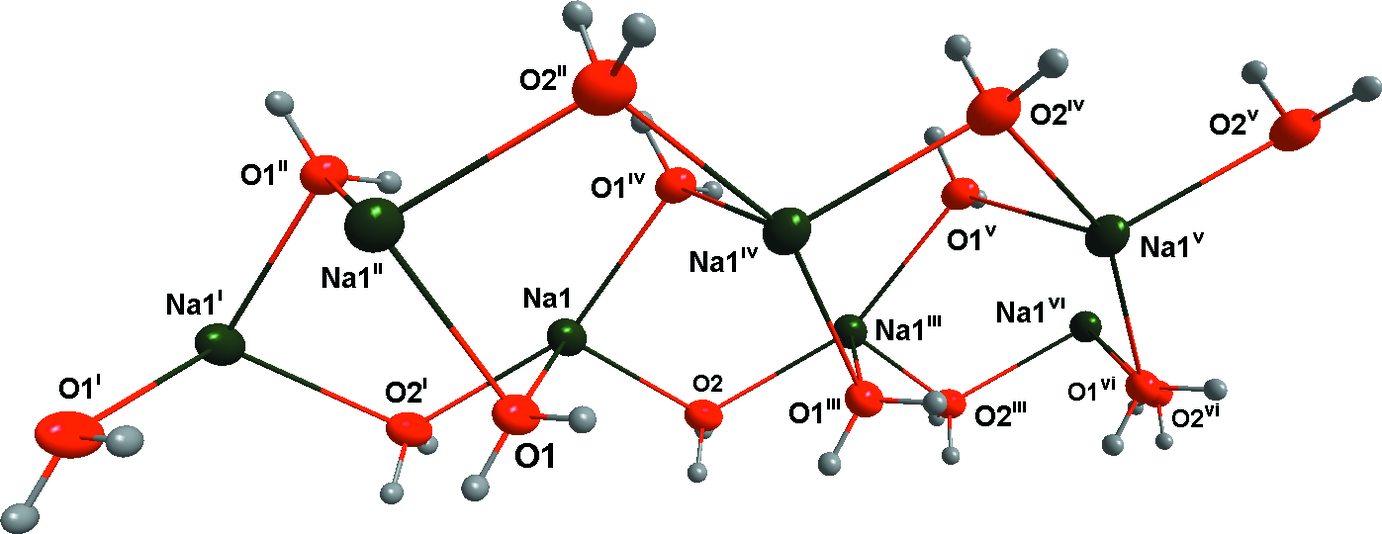

Supplement: Supplementary file 4 [file e-71-0m102-fig2.tif]

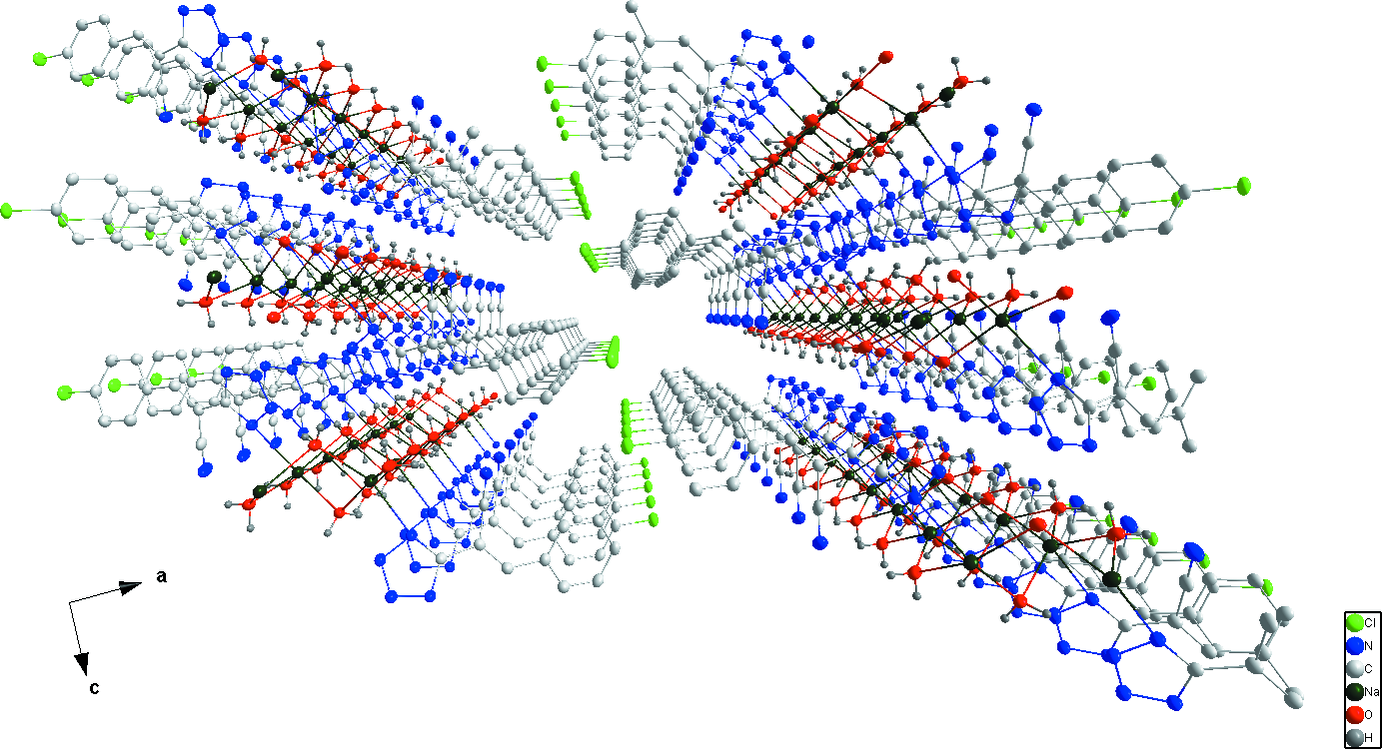

Supplement: Supplementary file 5 [file e-71-0m102-fig3.tif]

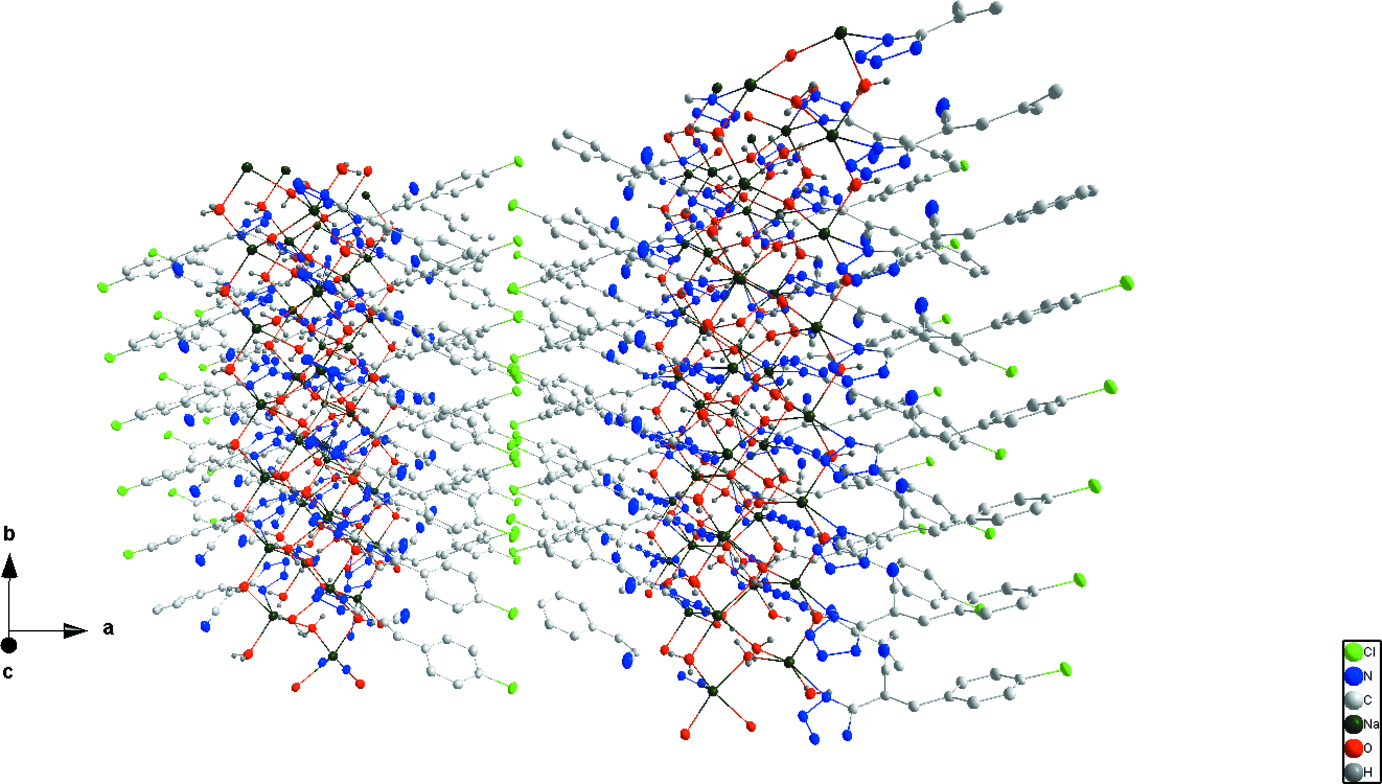

Supplement: Supplementary file 6 [file e-71-0m102-fig4.tif]
